# Theoretical Studies of the Interaction between Screw Surface and Material in the Mixer

**DOI:** 10.3390/ma14040962

**Published:** 2021-02-18

**Authors:** Andrzej Marczuk, Vasily Sysuev, Alexey Aleshkin, Petr Savinykh, Nikolay Turubanov, Andrzej Tomporowski

**Affiliations:** 1 Department of Agricultural, Forestry and Transport Machines, Faculty of Production Engineering, University of Life Sciences in Lublin, 20-950 Lublin, Poland; andrzej.marczuk@up.lublin.pl; 2 Laboratory of Livestock Mechanization, Federal State Budgetary Scientific Institution “Federal Agrarian Scientific Center of the North-East Named after N. V. Rudnitsky”, Kirov 610007, Russia; sisuev@mail.ru (V.S.); aleshkin.a.v@mail.ru (A.A.); peter.savinyh@mail.ru (P.S.); nikolaytu@mail.ru (N.T.); 3 Department of Theoretical and Structural Mechanics, Vyatka State University, Kirov 61000, Russia; 4 Department of Machines and Technical Systems, Faculty of Mechanical Engineering, University of Science and Technology in Bydgoszcz, 85-796 Bydgoszcz, Poland

**Keywords:** mixer, mixed feed, theoretical studies, power consumption, mixture components

## Abstract

Mixing is one of the most commonly used processes in food, animal feed, chemical, cosmetic, etc., industries. It is supposed to provide high-quality homogenous, nutritious mixtures. To provide appropriate mixing of materials while maintaining the process high efficiency and low energy consumption it is crucial to explore and describe the material flow caused by the movement of mixing elements and the contact between particles. The process of mixing is also affected by structural features of the machine components and the mixing chamber, speed of mixing, and properties of the mixed materials, such as the size of particles, moisture, friction coefficients. Thus, modeling of the phenomena that accompany the process of mixing using the above-listed parameters is indispensable for appropriate implementation of the process. The paper provides theoretical power calculations that take into account the material speed change, the impact of the material friction coefficient on the screw steel surface and the impact of the friction coefficient on the material, taking into account the loading height of the mixing chamber and the chamber loading value. Dependencies between the mixer power and the product degree of fineness, rotational speed of screw friction coefficients, the number of windings per length unit, and width of the screw tape have been presented on the basis of a developed model. It has been found that power increases along with an increase in the value of these parameters. Verification of the theoretical model indicated consistence of the predicted power demand with the power demand determined in tests performed on a real object for values of the assumed, effective loading, which was 65–75%.

## 1. Introduction

In order to increase the efficiency of livestock production, it is necessary to use food balanced in nutritional value. The main step in the preparation of balanced feeds is to mix the components [1,2,3,4]. One of the most commonly used in mixing processes are screw mixers (single, double and multi-screw) [5], to which the considerations in this paper will be limited.

Final product mixes must be highly homogeneous. Due to the variety of materials that are mixed with each other (they can be both bulk materials and liquid additives), it is necessary to carry out detailed theoretical considerations and, consequently, to verify developed models by means of experiment, in order to describe the processes of materials mixing to achieve the highest possible process efficiency and product quality [4,6,7,8]. The flow of materials, and the resulting mixing efficiency, depends on the parameters of the mixed materials (particle size, moisture, porosity, particle shape, friction coefficients) [9,10,11], and the design features (geometric, material) of the mixer [4,12,13] as well as the parameters of the mixing process itself, e.g., time, rotational speed of the mixing elements, the size of the stream of dosed materials, power and torques [14,15,16].

Incorrect selection of the design and process parameters results in disturbances in the mixing process and in obtaining an incomplete, heterogeneous product of low quality [17]. Disturbances in the mixing process cause other operational problems, e.g., increases in specific energy consumption, maintenance costs, damage to working elements and their faster wear, and difficulties in keeping the machine clean [1].

The need for theoretical modeling of mixing processes results precisely from the operational problems in the machine mixing and the problems related to the material flow itself in contact with the working elements of the mixers, which include, among others: loss of the mobility of the bulk material due to clogging of the dosing channels in the feed supply systems; too high intermolecular friction, which causes the effect of clogging of the material, and consequently leads to material losses or deterioration of the quality of the mixing product, and even energy losses and a decrease in the process efficiency and, thus, an increase in costs [9,18,19,20]. Therefore, it seems rational to model and search for optimal material, process and construction parameters for the bulk materials mixing processes, taking into account the physical basis of the behavior of these materials in contact with the working surface of the mixing devices.

The theoretical, analytical studies of the effect of structural and technological parameters on power consumption when mixing different feed components are rather rarely, because of complicated calculations, due to many factors affecting the process [21]. Therefore, a number of assumptions need to be used in calculations.

Experimental tests and theoretical studies of the mixing process of solutions are presented in work [22]. Among others, the impact of the feed material motion, caused by pumps and mixers, on energy efficiency of different structural features (geometric) of mixing units has been analyzed. Sulisz and Paprota [23] proposed a semi-analytical solution to be used for modeling of dependencies between the water particle motion and the mixture temperature changes. They have proven consistence of the theoretical model with the results of tests performed using laboratory mixing devices. George et al. [24] present the results of comparative and numerical analyses and experimental tests for determination of Rayleigh–Taylor mixing rates, which prove that the results of theoretical, experimental and numerical calculations of the acceleration rate are consistent when the values of acceleration rate are renormalized with regard to mass diffusion. Theoretical research and experimental tests have proven that the properties of materials motion and mixing largely depend on the grain moisture and when modeling the material flow it is necessary to know the particle size and shape distributions [9,25]. Most of theoretical models were associated with calculation of torques, energy consumption and volumetric efficiency. Roberts [26] developed a model of volumetric performance taking into account the so called granular Vortex motion, which depends on internal friction and friction between the particles and helical surface. Additionally, models based on the particle diameter and the transported mass volume were used for theoretical descriptions of the capacity, whereas the motion of particles was determined as a resultant of the screw size and the friction angle between the particle and the screw surface [27]. Work [28] presents a discussion on the subject of the impact of structural solutions of a feeding screw on the power and loads during operation. It was indicated that a complex approach, that is, an analytical-experimental one is necessary in design and power prediction for conveyors. Dynamics based studies of the particle motion in contact with the screw surface are presented on the example of feeding screws by proving that the screw rotational speed and the material pressure inside the screw have an impact on the operational efficiency [29]. Theoretical analyses provided by the literature are based on assumptions which do not find application in small scale devices which do not account for interaction between the particles and the particles and the walls of the device [9]. In this work, complex analyses have been presented taking into account these interactions in modeling of a mixer power consumption. 

Calculations for mixing processes, due to the discrete nature and the multitude of factors affecting the course of the process, are performed on the basis of numerical methods and models, including the discrete elements method (DEM) [9,30]. For example, Pezo et. al. [21] investigated numerically (with the use of DEM) and experimentally the influence of various design forms of the screw mixers on the efficiency of the mixing process. Bednarek et. al. [31] created a DEM algorithm for the mixing process in a conical screw mixer, which allowed to shorten the computation time. Cai et al. [5] also used DEM to determine the effect of rotational speed and sweeping speed on process quality and mixing efficiency in a double-screw conical mixer. Similar studies conducted by Qi et al. [32], showed that the rotational speed of the screw does not significantly affect the mixing process (as opposed to Cai et. Al. [5]) while increasing pitch length and reducing solid feed rates causes decrease in mixing performance. Connelly and Kokini [33] in turn, using the 2D finite element method (FEM), determined the velocities and trajectories of particle flow in a single and double screw mixer. Similar studies, but using 3D FEM, were carried out by Rathod and Kokini [34]. Based on the numerical analysis with the use of DEM and the experimental results, Pezo et al. [35] determined a mathematical model in the form of second order polynomial and artificial neural network model describing the quality of mixing in a double-screw mixer. In the paper [36], Mihailova et al. demonstrated using computational fluids dynamics (CFD) that mixer height is a key parameter influencing mixing performance. The influence of some design features of mixing elements on mixing efficiency in cylindrical mixer with use of DEM was presented in [37] showing, that three bladed mixer has better performance that mixer with two or four blades. The attempts have also been undertaken to experimentally determine the mixing process parameters. Kingston and Heindel [16] determined experimentally optimal process parameters of mixing in a screw-mixer, indicating that the best process efficiency occurs at a rotational speed of 60 rpm and dimensionless screw pitch of 1.75. In other work [25], the effect of scale on mixing effectiveness was investigated. The screw rotation speed, screw rotation orientation, dimensionless screw pitch, and particle size were investigated and compared for three double-screw mixers of differing scales [25] and it was found that scaling up with smaller biomass particle sizes results in significantly greater losses in effectiveness than for larger biomass particles. In [38] it was shown that the smaller size of mixed particles, the highest efficiency of mixing, while reducing particles concentration causes the reduction in efficiency.

To date, numerous studies (some of them described in this introduction) have been conducted on the interaction between the material particles and the working bodies of mixers, but they do not fully reflect the processes occurring in the mixing chamber; therefore, our theoretical studies presented here are relevant.

According to the presented motivation, the purpose of this work is to study the interaction between the screw surface of the mixer screw and the material.

The contributions of this paper include:

- Dynamic and kinematic analysis of flow of granular material in the mixer including interaction between screw surface and material,
- Determination of the influence of degree of grinding of the material, speed of mixer shaft rotation, density of material, coefficient of friction of material against steel and material, number of screw turns per unit length and width of the screw tape on the mixer power value, described with mathematical dependence,
- The determination of ranges of optimal design parameters of the screw mixer and mixing process based on the conducted theoretical study.

## 2. Material and Methods

**Figure 1:**
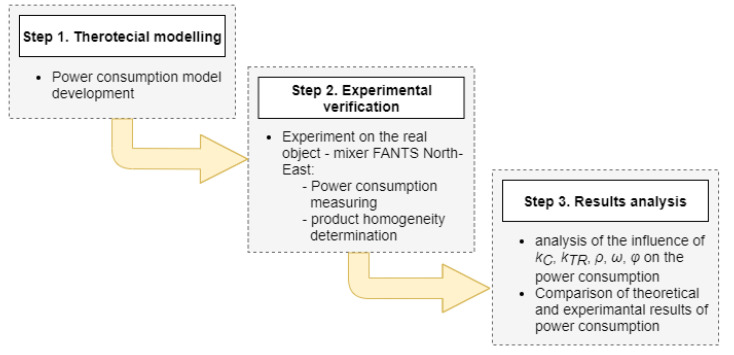
A scheme of procedures used in the study, *k_C_* is the coefficient of friction between material grains; *k_TR_* is the coefficient of friction between material and steel; *ρ* is the screw radius; *ω* is the screw angular speed; *φ* is the angle of interaction of screw with the material.

The major element of this work is theoretical research concerning the contact between the mixed material and the mixing elements and the relations of mutual contacts between the particles caused by mixing. The theoretical considerations were completed with verification of the power models using a real object. In Figure 1, there is a scheme of procedures for the accomplishment of the undertaken task.

### 2.1. Research Object

**Figure 2:**
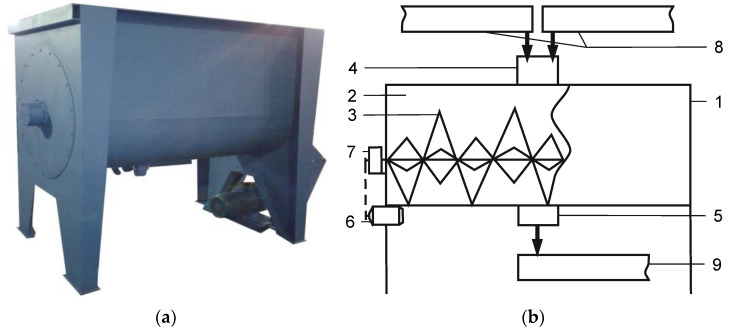
Mixer: (**a**) general view; (**b**) structural-technological scheme: 1—body; 2—mixing chamber, 3—mixer; 4—filling chamber; 5—discharge chamber; 6-engine; 7—reductor; 8—supply screw feeder; 9—discharge screw feeder.

The theoretical analyses of the interaction between the screw surface and material in the mixer as well as the physical experiment were carried out for a horizontal screw mixer manufactured by FANTS North-East [39]. The mixer consists of a housing, mixing chamber, mixing element, filling chamber, discharge chamber, engine and power transmission (Figure 2).

**Figure 3:**
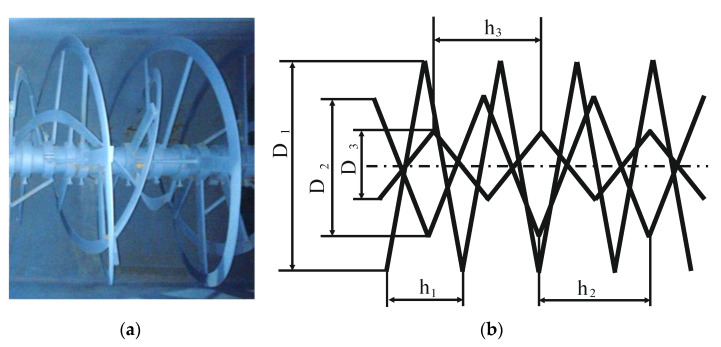
The stirrer of a screw mixer: (**a**) general view; (**b**) scheme: *D*_1_—diameter of external screw; *D*_2_—diameter of middle screw; *D*_3_—diameter of internal screw; *h*_1_—pitch of external screw; *h*_2_—pitch of middle screw; *h*_3_—pitch of internal screw.

The main element of the mixer is a stirrer that is a special structure, shown in Figure 3. The structure of a stirrer consists of three ribbons that make up a screw line with different diameters *D* and winding pitch *h*. The geometric structural parameters of the stirrer are presented in Table 1.

### 2.2. Assumptions of Theoretical Analyses

**Figure 4:**
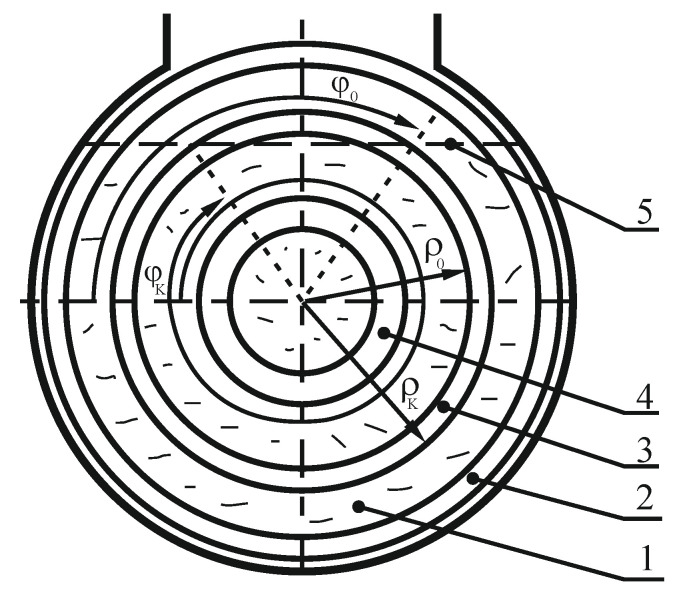
Scheme of the mixing chamber with marked interaction angles *φ* and central screw radii *ρ*; 1—mixing chamber; 2—external screw; 3—middle screw; 4—internal screw; 5—upper material layer of the mixing chamber.

When carrying out theoretical calculations, the analytical mechanical methods were used. Figure 4 presents the layout of the mixing chamber accepted for calculations. The figure presents the initial (*φ*_0_) and final (*φ*_K_) angles of the central screw interaction with the material, (angle *φ* is measured from the level and has different values for each screw for the same amounts of material) and initial (*ρ*_0_) and final (*ρ_K_*) radii of the central screw. The values of screw parameters presented in Table 1 were assumed for calculations. The screw angular speed range accepted for the research was 1.05–4.19 rad∙s^−1^.

The research covered physical properties of the materials: specific density γ, coefficient of material against material friction *k_C_* and steel *k_TR_* at a rest and during motion. Physical properties of the mixed materials used for preparation of fodder mixtures depend on the size of comminuted particles. The materials property ranges, in which the minimal and maximal values determine the range of properties of all materials used for the production of fodder mixtures, were accepted for calculations [40,41] (Table 2).

### 2.3. Conditions of Experimental Tests

**Figure 5:**
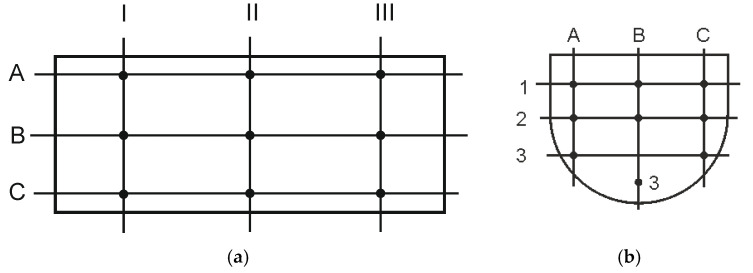
Sites the samples were collected for determination of the product homogeneity coefficient after mixing: (**a**) in the horizontal plane; (**b**) in the vertical plane.

A FANTS North-East [39] mixer was used in verification of experimental tests. In the tests a mixture of barley (80% of base) and rye (20% of base, 88% of the mixture total mass) was used and peas as a control component (12% of total mass of mixture), with properties given in Table 2. Mixing time, excluding loading time, was 10 min [7], and the stirrer angular speed was 2.25 rad∙s^−1^. The mixer loading level was accepted to be a variable referred to as the amount of material in the mixing chamber as a percentage of the maximum load. The 45% of the chamber loading was accepted to be the initial value, and it was changed by 10% until achievement of the maximal value 95%. Three repetitions were performed for each loading level of mixing chamber, each time recording the power consumption and the product non-homogeneity degree. Next, the arithmetic mean of the measured values was calculated for each case. Power consumption was recorded by means of a wattmeter. Power consumption was also measured for the mixer idle run. In order to determine homogeneity of the product of mixing, after each experiment samples were collected from different parts of the mixing chamber (in total 27 samples) according to the scheme presented in Figure 5.

Next, the samples were sieved on a sieve screen to separate the control fractions (peas) from the base and based on this, the peas weight was determined for each sample. The product variability (non-homogeneity) coefficient was successively determined according to equation:

(1) $$V_{c}=\frac{\sqrt{\frac{\Sigma {\left(x_{i}-\bar{x}\right)}^{2}}{n-1}}}{\bar{x}}\cdot 100\%,$$

where *x_i_* is the current, observed value; $\bar{x}$ is the arithmetical mean of the observed value; $\bar{x}=\left(\sum x_{i}\right)/n$; *n* is the number of observations (samples).

(2) $$\nu =100-V_{c}.$$

Next, the result of the mixer power consumption tests in dependence on the chamber loading level was presented with the results of power calculations for the materials and process parameters consistent with those applied in experimental tests. 

## 3. Theoretical Approach

**Figure 6:**
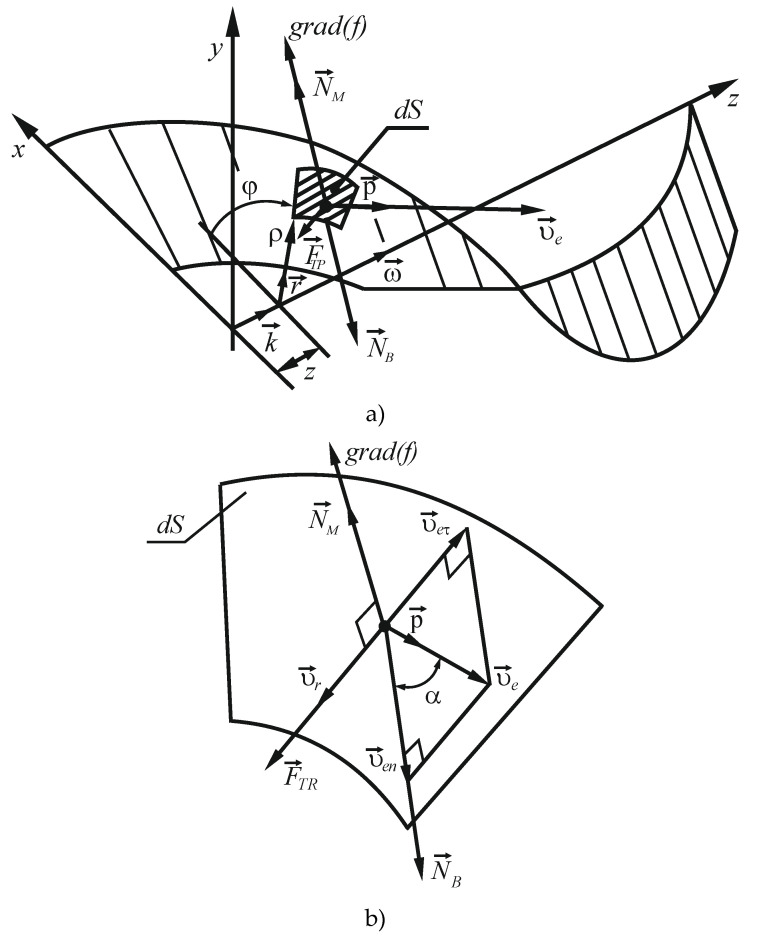
Schematic representation of interaction between the screw surface and the material in the mixer: (**a**) axes of coordinates and forces, (**b**) elementary area—a segment of the screw surface and material rate; *grad* (*f*) is the gradient vector to the surface *f*; $\vec{r}$, $\vec{p},\;\vec{k}$ are the cylindrical unitary vectors; *dS* is the elementary area; ${\vec{N}}_{B}$ is the force with which an elementary area of the screw surface impacts the material when in steady motion; ${\vec{N}}_{M}$ is the normal force; $\vec{\omega }$ is the angular velocity; ${\vec{v}}_{e}$ is the transfer velocity; ${\vec{v}}_{r}$ is the relative velocity; *φ* is the angle of screw interaction with material and *ρ* is the screw radius.

Let us consider the rotation of the screw (helical) surface around its axis in a horizontal mixer for bulk materials using the theorem on the change of angular (kinetic) momentum [42]:

(3) $$I\frac{d\omega }{dt}=M_{VR}+\sum (N_{M\varphi }\rho +F_{TR\varphi }\rho ),$$

where I is inertia moment of the mixer shaft together with the screw surface and material moved in transfer motion, ω is angular velocity of the shaft, $M_{VR}$ is torque moment from the drive applied to the shaft, $N_{M\varphi }$ is normal material response acting on the elementary area of the screw surface in the projection onto the direction of its motion, that is, on the cylindrical axis $\vec{p}$, $F_{TR\varphi }$ is the material friction force applied to the elementary area of the screw surface in projection onto the axis $\vec{p}$, ρ is the radius in the cylindrical coordinates of the elementary area of the surface, which is the arm of the component forces along the direction $\vec{p}$, $\sum (N_{M\varphi }\rho +F_{TR\varphi }\rho )$ means the summation of moments from forces applied to all elementary areas of the screw surface (Figure 6).

For dynamic analysis of interaction between the material and the screw surface, we assume that gravity forces applied to the mixed material are symmetric relative to the vertical axial section of the mixer, and that they do not affect the rotation of the screw. The second assumption is that in the process of interaction between the elementary volume of the material and the elementary area of the screw surface, the friction forces from other elementary volumes of the material are negligible.

When the mixer shaft rotates at a constant angular velocity ($\frac{d\omega }{dt}=0$), the moment required to maintain it, without considering structural friction resistance, can be determined as per Equation (3):

(4) $$M_{VR}=-\sum (N_{M\varphi }\rho +F_{TR\varphi }\rho ).$$

Normal response ${\vec{N}}_{M}$ is directed along the gradient towards the screw surface. The equation of the screw surface in cylindrical coordinates is written as:

(5) f(ρ,φ,z)=z−aφ=0.

Gradient vector to the surface f(ρ,φ,z) has the following components according to the cylindrical unitary vectors ($\vec{r},\vec{p},\vec{k})$:

(6) $$grad\left(f\right)=\frac{\partial f}{\partial \rho }\vec{r}+\frac{1}{\rho }\frac{\partial f}{\partial \varphi }\vec{p}+\frac{\partial f}{\partial z}\vec{k}.$$

Partial derivatives in Equation (6) are equal to:

(7) $$\{\begin{array}{c} \frac{\partial f}{\partial \rho }=0;\\ \frac{\partial f}{\partial \varphi }=-a;\\ \frac{\partial f}{\partial z}=1. \end{array}$$

Then the gradient to the surface is

(8) $$grad\left(f\right)=-\frac{a}{\rho }\vec{p}+1\cdot \vec{k}.$$

Its modulus is:

(9) $$\left|grad\left(f\right)\right|=\sqrt{{\left(\frac{a}{\rho }\right)}^{2}+1}.$$

For normal response ${\vec{N}}_{M}$, projections are proportional to projections of the gradient vector on the cylindrical coordinate axes:

(10) $$\{\begin{array}{c} N_{M\rho }=0;\\ N_{M\varphi }=-\lambda \frac{a}{\rho };\\ N_{Mz}=\lambda \;, \end{array}$$

where λ is an indefinite Lagrange multiplier.

When using the right screw of the surface in accordance with Equation (5) and the angular velocity $\vec{\omega }$ is co-directional with the z axis (see Figure 6), this factor is positive (λ≥0). The magnitude of the force ${\vec{N}}_{M}$, determines the friction force, ${\vec{F}}_{TR}$, in the modulus in accordance with the Coulomb’s law for dry sliding friction with the coefficient of $k_{TR}$: (*λ* ≥ 0).

(11) $$\left|{\vec{F}}_{TR}\right|=k_{TR}\left|{\vec{N}}_{M}\right|=k_{TR}\lambda \sqrt{{\left(\frac{a}{\rho }\right)}^{2}+1}.$$

The direction of the friction force ${\vec{F}}_{TR}$ is opposite to relative velocity of the elementary area of the screw surface relative to the material ${\vec{v}}_{\tau }$. The mixed material is at a standstill before it interacts with the screw surface. After interaction, it acquires a velocity that has two components: transfer $\vec{v_{e}}$, and relative velocity in motion along the screw surface ${\vec{v}}_{r}$, and friction force applied to the latter coincides in direction with the elementary surface:

(12) $${\vec{F}}_{TR}=k_{TR}\left|{\vec{N}}_{M}\right|\frac{{\vec{v}}_{r}}{\left|{\vec{v}}_{r}\right|}.$$

We assume that after interaction with the screw surface the material slides in its tangent plane, maintaining the value of the relative velocity component, which it had before the interaction. Since the material was standstill before the interaction, the relative velocity ${\vec{v}}_{r}$ is equal to ${\vec{v}}_{e\tau }$—the projection of velocity of the elementary area $\vec{v_{e}}$ onto the tangent plane to the surface, but is opposite in direction (as was presented in Figure 6b):

(13) $${\vec{v}}_{r}=-{\vec{v}}_{e\tau }.$$

Let us define the projection of velocity $\vec{v_{e}}$ onto the direction of the gradient vector:

(14) $$v_{en}=\frac{\vec{v_{e}}\cdot grad\left(f\right)}{\left|grad\left(f\right)\right|}=\;\frac{v_{e}\cdot \left(-\frac{a}{\rho }\right)}{\sqrt{{\left(\frac{a}{\rho }\right)}^{2}+1}}.$$

Then the magnitude of the tangential velocity component is found by the Pythagorean theorem:

(15) $$v_{e\tau }=\sqrt{{v_{e}}^{2}-{v_{e}}^{2}}=v_{e}\frac{1}{\sqrt{{\left(\frac{a}{\rho }\right)}^{2}+1}}.$$

Taking into account Equation (13), the modulus of relative velocity $v_{r}$ is equal to the value $v_{e\tau }$:

(16) $$v_{r}=v_{e}\frac{1}{\sqrt{{\left(\frac{a}{\rho }\right)}^{2}+1}}.$$

Let us find the projections of the elementary surface velocity $v_{e\tau }$ onto the cylindrical coordinate axes, taking into account the vector equality:

(17) $${\vec{v}}_{e\tau }={\vec{v}}_{e}-{\vec{v}}_{en}.$$

The projections of vector ${\vec{v}}_{e}$ on the right-hand side of Equation (17) take the form:

(18) $$\{\begin{array}{c} v_{e\rho }=0;\\ v_{e\varphi }=v_{e}=\omega \rho ；\\ v_{ez}=0. \end{array}$$

The projections of the normal component of the elementary surface velocity ${\vec{v}}_{en}$ onto the coordinate axis are determined taking into account its collinearity with the vector gradient:

(19) $$v_{en\rho }={\vec{v}}_{en}\cdot \vec{r}=v_{en}\frac{{\left(grad\left(f\right)\right)}_{\rho }}{\left|grad\left(f\right)\right|}=0;$$

(20) $$v_{en\varphi }={\vec{v}}_{en}\cdot \vec{p}=v_{en}\frac{{\left(grad\left(f\right)\right)}_{\varphi }}{\left|grad\left(f\right)\right|}=\frac{v_{e}\cdot \left(-\frac{a}{\rho }\right)}{\sqrt{{\left(\frac{a}{\rho }\right)}^{2}+1}}\cdot \frac{\left(-\frac{a}{\rho }\right)}{\sqrt{{\left(\frac{a}{\rho }\right)}^{2}+1}}=\frac{v_{e}\cdot {\left(\frac{a}{\rho }\right)}^{2}}{{\left(\frac{a}{\rho }\right)}^{2}+1};$$

(21) $$v_{enz}=v_{en}\frac{{\left(grad\left(f\right)\right)}_{z}}{\left|grad\left(f\right)\right|}=\frac{v_{e}\cdot \left(-\frac{a}{\rho }\right)}{\sqrt{{\left(\frac{a}{\rho }\right)}^{2}+1}}\cdot \frac{1}{\sqrt{{\left(\frac{a}{\rho }\right)}^{2}+1}}=-\frac{v_{e}\frac{a}{\rho }}{{\left(\frac{a}{\rho }\right)}^{2}+1}.$$

Then, by projecting Equation (17) onto the cylindrical axes, we obtain:

(22) $$v_{e\tau \rho }=v_{e\rho }-v_{en\rho }=0;$$

(23) $$v_{e\tau \varphi }=v_{e\varphi }-v_{en\varphi }=v_{e}-\frac{v_{e}\cdot {\left(\frac{a}{\rho }\right)}^{2}}{{\left(\frac{a}{\rho }\right)}^{2}+1}=v_{e}\frac{1}{{\left(\frac{a}{\rho }\right)}^{2}+1};$$

(24) $$v_{e\tau z}=v_{ez}-v_{enz}=0-\left(-\frac{v_{e}\cdot \frac{a}{\rho }}{{\left(\frac{a}{\rho }\right)}^{2}+1}\right)=\frac{v_{e}\cdot \frac{a}{\rho }}{{\left(\frac{a}{\rho }\right)}^{2}+1}.$$

Taking into account Equation (13), which reflects the motion of the screw surface in the initially motionless material, we find the projections of relative velocity ${\vec{v}}_{r}$ on the axis of the cylindrical coordinate system:

(25) $$\{\begin{array}{c} v_{r\rho }=0;\\ v_{r\varphi }=-v_{e}\frac{1}{{\left(\frac{a}{\rho }\right)}^{2}+1};\\ v_{rz}=-\frac{v_{e}\cdot \frac{a}{\rho }}{{\left(\frac{a}{\rho }\right)}^{2}+1}. \end{array}$$

We substitute Expressions (25) and (16) into Equation (12) and find the direction of the friction force applied to the elementary area:

(26) $$\{\begin{array}{c} F_{TR\rho }=0;\\ F_{TR\varphi }=-\frac{k_{TR}\left|{\vec{N}}_{M}\right|}{\sqrt{{\left(\frac{a}{\rho }\right)}^{2}+1}};\\ F_{TRz}=-\frac{k_{TR}\left|{\vec{N}}_{M}\right|}{\sqrt{{\left(\frac{a}{\rho }\right)}^{2}+1}}. \end{array}$$

If we know the projection of forces acting upon the elementary surface area, based on Equations (10) and (26) we can write down on the right-hand side of Equation (4):

(27) $$N_{M\varphi }\rho +F_{TR\varphi }\rho =-\lambda a-k_{TR}\lambda \rho .$$

The factor λ is equal to the ratio of the modulus of force ${\vec{N}}_{M}$—impact of the material on the area dS to the modulus grad(f)—gradient vector to the surface:

(28) $$\lambda =\frac{\left|{\vec{N}}_{M}\right|}{\sqrt{{\left(\frac{a}{\rho }\right)}^{2}+1}}.$$

In order to determine the value of ${\vec{N}}_{M}$, we consider the force ${\vec{N}}_{B}$ with which an elementary area of the screw surface impacts the material when in steady motion. According to Newton’s third law, these forces are equal in magnitude and opposite in direction:

(29) $${\vec{N}}_{M}=-{\vec{N}}_{B}.$$

**Figure 7:**
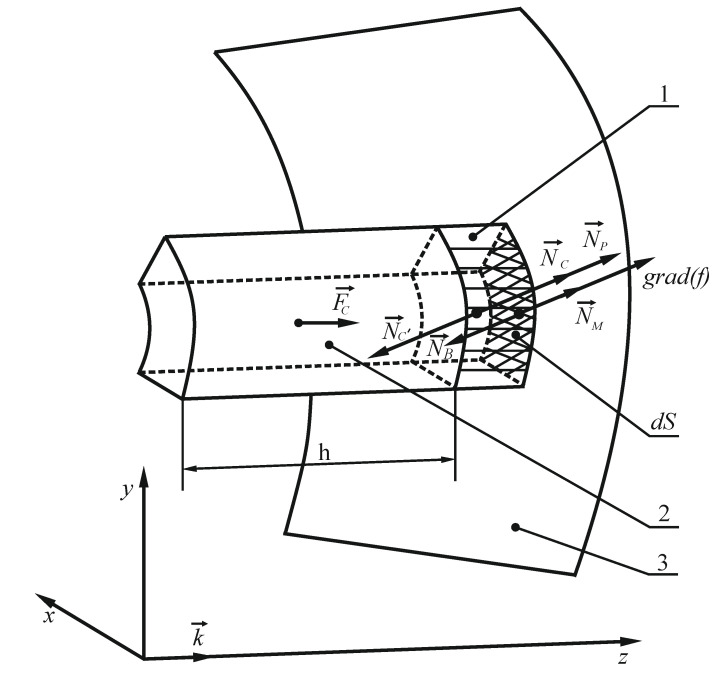
Schematic representation of interaction between the flow of accelerated material 1, which is in contact with the elementary area of the screw surface *dS*, and the horizontal layer 2 in the form of an oblique cylinder with a base *dS* and a height equal *h*, the screw pitch (balanced gravity forces and responses are not shown); *grad* (*f*), the gradient vector to the surface *f*; $\vec{N_{P}}$, the pressure applied by the upper layers of the material on the volume interacting with the elementary area dS; ${\vec{N}}_{M}$, normal force; ${\vec{N}}_{B}$, the force with which an elementary area of the screw surface impacts the material when in steady motion; $\vec{N_{C}}$, the force that overcomes the friction force $\vec{F_{C}}$.

Let us apply the theorem on the change in momentum to the volume of the material that interacts with the elementary area dS during a certain time period t in the projection onto the gradient vector to the surface. Taking into account that the fragment of material interacting with the surface is not a free flow, adjacent fragments act on it, in addition to the screw surface. We will conventionally group them into two components: $\vec{N_{P}}$ is the force due to pressure of the overlying layers of the material, and $\vec{N_{C}}$ is the force of normal pressure due to resistance to horizontal movement of the portion of the material enclosed between the screw turns (flights) and resting on the platform dS (see Figure 7). This load of material moves horizontally along the screw axis and acts on the flow adjacent to the surface, for which we write down:

(30) $$Q_{n}-Q_{n0}=-\int_{0}^{t}N_{B}dt+\int_{0}^{t}N_{P}+\int_{0}^{t}N_{C},$$

where $Q_{n}$ is the momentum acquired by the material in projection onto the gradient direction, $Q_{n0}$ is the initial momentum of motion of the material in the projection onto the gradient direction, $Q_{n0}=0$, since the material was at a standstill.

On the right-hand side of Equation (30), the impulse of force ${\vec{N}}_{B}$ is projected onto the direction of the gradient to the surface.

**Figure 8:**
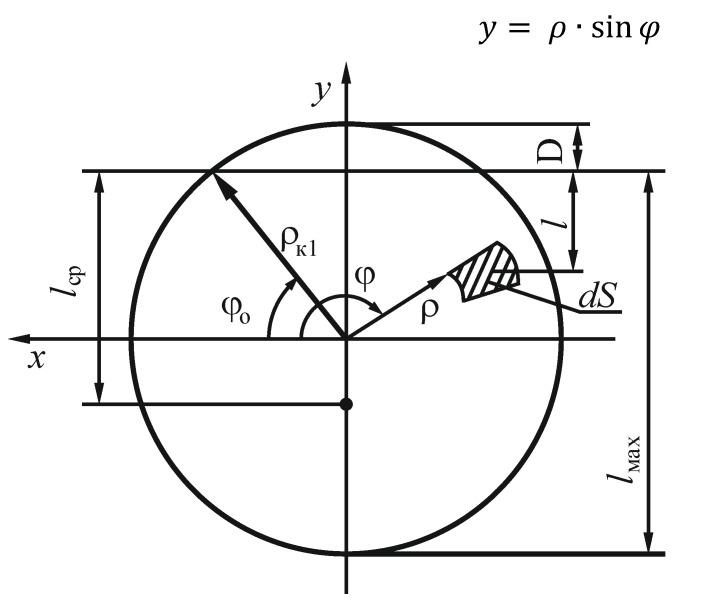
Schematic representation of the mixer fill-up and thickness of the material layer *l* to the location of the elementary area of the screw surface *dS*; *D* is the vertical height of the mixer ullage, $\rho_{к1}$ is maximum radius of external screw of the mixer, *φ* is the angle of screw interaction with material, $l_{cp}$ is the averaged depth of the material layer over the screw surface *S*; $l_{max}$ is the the maximal depth of the material layer over the screw surface *S*; *φ*_0_ is the initial angle of screw interaction of screw with material; and *ρ* is the screw radius.

Force $\vec{N_{P}}$ is the pressure applied by upper layers of the material on the volume interacting with the elementary area dS (Figure 7). It is determined by analogy with the pressure in a liquid, but on condition that the moving surface provides compressive stresses to the considered volume of bulk material (that is, there is no pressure from the rear side of the screw surface). Force $\vec{N_{P}}$ acts as the force of static pressure of the upper layers on the lower ones, assuming equality in all directions, similar to Pascal’s law for liquids:

(31) $$N_{P}=p\cdot dS,$$

where an analogue of the static pressure p is determined using the bulk density of the material γ, free fall acceleration g, and height l of overlying layers above the elementary area:

(32) $$p=\gamma \cdot g\left(\rho_{к1}-D-y\right),$$

where $\rho_{к1}$ is maximum radius of external screw of the mixer, D is vertical height of the mixer ullage D= $\rho_{к1}\left(1-sin\varphi_{0}\right)$, and y is a vertical Cartesian coordinate measured from the screw axis (interpretation of above parameters was presented in Figure 8):

(33) y= ρ·sinφ

Then:

(34) $$N_{P}=\gamma \cdot g\left(\rho_{к1}-D-\;\rho \cdot \sin\varphi \right)dS.$$

In Equation (30), force $\vec{\;N_{C}}$ is the force that overcomes friction force $\vec{\;F_{C}}$ (along the *z* axis), with horizontal movement of the material enclosed between the screw turns, with a length equal to the screw pitch h:

(35) $$\;F_{C}=g\cdot \gamma \cdot dS\cdot \cos\left(\hat{grad\left(f\right)\vec{k}}\right)\cdot h\cdot k_{c},)$$

where $k_{c}$ is the coefficient of friction between layers of the material, $dS\cdot \cos\left(\hat{grad\left(f\right)\vec{k}}\right)$ is the projection of the area dS onto the plane (x,y).

The total frictional force of the material moved by the screw surface along the horizontal axis z is equal to:

(36) $$\sum \;F_{C}=\iint_{\left(S\right)}g\cdot \gamma \cdot \cos\left(\hat{grad\left(f\right)\vec{k}}\right)\cdot h\cdot k_{c}dS.$$

However, this force is distributed unevenly along the vertical, since the lower layers are under pressure from the upper ones. Let us assume that the friction force during horizontal movement along the z axis is directly proportional to the layer depth along the y axis, so let us denote it by l:

(37) $$l=\left(\rho_{к1}-D-y\right),$$

then the friction force related to the elementary area dS is equal to:

(38) $$\;F_{C}=g\cdot \gamma \cdot dS\cdot \cos\left(\hat{grad\left(f\right)\vec{k}}\right)\cdot h\cdot k_{c}\frac{l}{l_{cp}},$$

where $l_{cp}$ is the averaged depth of the material layer over the screw surface *S*:

(39) $$l_{cp}=\frac{\iint_{\left(S\right)}ldS}{\iint_{\left(S\right)}dS}=\frac{\iint_{\left(S\right)}\left(\rho_{к1}-D-y\right)dS}{S},$$

where $l_{cp}$ is a constant value for steady motion, and it is defined as the ratio of integrals over a surface of the first kind:

(40) $$l_{cp}=\frac{\int_{\varphi_{0}}^{\varphi_{k}}\left(\int_{\rho_{0}}^{\rho_{к}}\;\left(\rho_{к1}-D-\;\rho \cdot \sin\varphi \right)\sqrt{a^{2}+\rho^{2}}d\rho \right)d\varphi }{\int_{\varphi_{0}}^{\varphi_{k}}\left(\int_{\rho_{0}}^{\rho_{к}}\sqrt{a^{2}+\rho^{2}}d\rho \right)d\varphi }.$$

The ratio between the friction force $\vec{\;F_{C}}$ and the force of additional normal pressure $\vec{\;N_{C}}$ on the elementary volume interacting with the screw surface is determined based on the assumption that motion of the material along the axis z is steady (uniform). In addition, the work of force $\vec{N{}^{\prime }{}_{C}}$ along possible motion δz is equal in magnitude and opposite in sign to the work of the force $\vec{\;F_{C}}$ along the same motion (see Figure 7, the material moves opposite to the z axis):

(41) $$-N{}^{\prime }{}_{C}\cdot \delta z\cdot \cos\left(\hat{grad\left(f\right)\vec{k}}\right)+\;F_{C}\cdot \delta z=0.$$

Force $\vec{\;N{}^{\prime }{}_{C}}$ correlates with force $\vec{\;N_{C}}$ according to action-reaction phenomenon:

(42) $$\vec{N{}^{\prime }{}_{C}}=-\vec{\;N_{C}},$$

where $\cos\left(\hat{grad\left(f\right)\vec{k}}\right)=\frac{1}{\sqrt{{\left(\frac{a}{\rho }\right)}^{2}+1}}$ is the angle cosine between the gradient to the surface and the z axis. Then:

(43) $$\;N_{C}=\frac{\;F_{C}}{\cos\left(\hat{grad\left(f\right)\vec{k}}\right)}=\gamma \cdot dS\cdot h\cdot k_{c}\frac{l}{l_{cp}}=g\cdot \gamma \cdot dS\cdot h\cdot k_{c}\frac{\left(\rho_{к1}-D-y\right)}{l_{cp}}.$$

When we substitute the expression for y, we obtain:

(44) $$\;N_{C}=g\cdot \gamma \cdot h\cdot k_{c}\frac{\left(\rho_{к1}-D-\rho \cdot \sin\varphi \right)}{l_{cp}}\cdot dS$$

After interacting with the surface, the material acquires velocity $\vec{v}$, which is made up of the transfer ${\vec{v}}_{e}$ and relative ${\vec{v}}_{r}$ components, the projections of which on the coordinate axes were previously determined by Equations (18) and (25), respectively. The relative velocity ${\vec{v}}_{r}$ has no projection onto the gradient vector, while the transfer velocity ${\vec{v}}_{e}$ has a projection onto the gradient direction according to Equation (14), then:

(45) $$Q_{n}=m\cdot \frac{v_{e}\cdot \left(-\frac{a}{\rho }\right)}{\sqrt{{\left(\frac{a}{\rho }\right)}^{2}+1}},$$

where m is mass of the material interacting with an elementary area in t time. With steady motion, when the screw surface is immersed in the material, the value m is determined by the expression:

(46) $$m=\gamma \cdot v_{e}\cdot t\cdot dS\cdot \cos\left(\alpha \right),$$

where γ is bulk density of the material, α is the angle between the motion direction of the area dS and the direction opposite to the vector-gradient of this area. The direction is opposite because the screw interacts with the material with the side opposite to the direction grad(f) (see Figure 6):

(47) $$\cos\left(\alpha \right)=\vec{p}\cdot \frac{\left(-grad\left(f\right)\right)}{\left|grad\left(f\right)\right|}=\frac{\frac{a}{\rho }}{\sqrt{{\left(\frac{a}{\rho }\right)}^{2}+1}}.$$

Then, after substituting Equations (47) into (46) and (46) into (45), we obtain:

(48) $$Q_{n}=-\gamma \cdot {v_{e}}^{2}\cdot t\cdot dS\frac{{\left(\frac{a}{\rho }\right)}^{2}}{{\left(\frac{a}{\rho }\right)}^{2}+1}.$$

We compose the theorem on the change in the momentum (Equation (30)) with steady motion, and we obtain:

(49) $$-\gamma \cdot {v_{e}}^{2}\cdot t\cdot dS\frac{{\left(\frac{a}{\rho }\right)}^{2}}{{\left(\frac{a}{\rho }\right)}^{2}+1}-0=-N_{B}\cdot t+N_{P}\cdot t+N_{C}\cdot t.$$

Hence, we determine the magnitude of the force of action of an elementary area dS on the material:

(50) $$\begin{array}{c} N_{B}=\gamma \cdot {v_{e}}^{2}\cdot dS\frac{{\left(\frac{a}{\rho }\right)}^{2}}{{\left(\frac{a}{\rho }\right)}^{2}+1}+\gamma \cdot g\left(\rho_{к1}-D-\;\rho \cdot \sin\varphi \right)dS\;+\\ +g\cdot \gamma \cdot h\cdot k_{c}\frac{\left(\rho_{к1}-D-\rho \cdot \sin\varphi \right)}{l_{cp}}\cdot dS. \end{array}$$

Taking into account Equations (50) and (29), we obtain the relation for algebraic value of the material impact force on the area dS:

(51) $$\begin{array}{c} N_{M}=N_{B}=\gamma \cdot {v_{e}}^{2}\cdot dS\frac{{\left(\frac{a}{\rho }\right)}^{2}}{{\left(\frac{a}{\rho }\right)}^{2}+1}+\gamma \cdot g\left(\rho_{к1}-D-\;\rho \cdot \sin\varphi \right)dS\;+\\ +g\cdot \gamma \cdot h\cdot k_{c}\frac{\left(\rho_{к1}-D-\rho \cdot \sin\varphi \right)}{l_{cp}}\cdot dS. \end{array}$$

Due to the awkwardness of Equation (51), the factor λ, which is the coefficient of proportionality between the gradient vector grad(f) and the force ${\vec{N}}_{M}$, is divided into three components in accordance with the components in Equation (51), by denoting:

(52) $$\lambda_{Q}=\frac{\gamma \cdot {v_{e}}^{2}\cdot dS\frac{{\left(\frac{a}{\rho }\right)}^{2}}{{\left(\frac{a}{\rho }\right)}^{2}+1}}{\left|grad\left(f\right)\right|};$$

(53) $$\lambda_{p}=\frac{\gamma \cdot g\left(\rho_{к1}-D-\;\rho \cdot \sin\varphi \right)dS}{\left|grad\left(f\right)\right|};$$

(54) $$\lambda_{c}=\frac{\gamma \cdot h\cdot k_{c}\frac{\left(\rho_{к1}-D-\rho \cdot \sin\varphi \right)}{l_{cp}}\cdot dS}{\left|grad\left(f\right)\right|};$$

(55) $$\lambda =\lambda_{Q}+\lambda_{p}+\lambda_{c}.$$

Based on the expression, the first component $\lambda_{Q}$ gives:

(56) $$\lambda_{Q}=\gamma \cdot {v_{e}}^{2}\cdot dS\frac{{\left(\frac{a}{\rho }\right)}^{2}}{{\left({\left(\frac{a}{\rho }\right)}^{2}+1\right)}^{\frac{3}{2}}}.$$

Using the factor $\lambda_{Q}$ in Equation (27), we obtain:

(57) $$\begin{array}{c} {\left(N_{M\varphi }\rho +F_{TR\varphi }\rho \right)}_{Q}=-\gamma \cdot {v_{e}}^{2}\cdot dS\frac{{\left(\frac{a}{\rho }\right)}^{2}\left(a+k_{TR}\rho \right)}{{\left({\left(\frac{a}{\rho }\right)}^{2}+1\right)}^{\frac{3}{2}}}=\\ =-\gamma \cdot \;\omega^{2}\cdot dS\frac{a^{2}\left(a+k_{TR}\rho \right)}{{\left({\left(\frac{a}{\rho }\right)}^{2}+1\right)}^{\frac{3}{2}}}. \end{array}$$

By substituting the result Equation (57) into Equation (4), we find the torque required to impact on the material at a given angular velocity ω:

(58) $$M_{VRQ}=\sum \gamma \cdot \;\omega^{2}\cdot \frac{a^{2}\left(a+k_{TR}\rho \right)}{{\left({\left(\frac{a}{\rho }\right)}^{2}+1\right)}^{\frac{3}{2}}}dS.$$

When we descend from discrete summation to continuous summation, we obtain an integral taken over the screw surface of the first kind:

(59) $$M_{VRQ}=\underset{\left(S\right)}{\iint }\gamma \cdot \;\omega^{2}\cdot \frac{a^{2}\left(a+k_{TR}\rho \right)}{{\left({\left(\frac{a}{\rho }\right)}^{2}+1\right)}^{\frac{3}{2}}}dS.$$

Considering that the area of an elementary area is:

(60) $$dS=\sqrt{a^{2}+\rho^{2}}d\rho d\varphi ,$$

we split the integral over the surface into a double multiple integral over two cylindrical coordinates:

(61) $$M_{VRQ}=\int_{\varphi_{0}}^{\varphi_{k}}\left(\int_{\rho_{0}}^{\rho_{к}}\gamma \cdot \;\omega^{2}\cdot \frac{a^{2}\left(a+k_{TR}\rho \right)}{{\left({\left(\frac{a}{\rho }\right)}^{2}+1\right)}^{\frac{3}{2}}}\sqrt{a^{2}+\rho^{2}}d\rho \right)d\varphi ,$$

where $\varphi_{0},\;\varphi_{k}$ are the initial and final values of the vector angle, and $\rho_{0},\rho_{k}$ are the initial and final values of the radius.

When we simplify the numerator and denominator of Equation (61) by $\sqrt{a^{2}+\rho^{2}}$, we obtain:

(62) $$M_{VRQ}=\int_{\varphi_{0}}^{\varphi_{k}}\left(\int_{\rho_{0}}^{\rho_{к}}\gamma \cdot \omega^{2}\cdot \frac{a^{2}\left(a+k_{TR}\rho \right)}{\left(a^{2}+\rho^{2}\right)}\rho^{3}d\rho \right)d\varphi .$$

Equation (62) has an awkward analytical expression, thus the calculations were carried out using Mathcad software (Mathsoft, Cambridge, MA, USA) using a symbolic processor and the subsequent substitution of the numerical values of all quantities.

Similarly, based on the second component in Equation (51), we obtain:

(63) $$\lambda_{P}=\gamma \cdot g\left(\rho_{к1}-D-\;\rho \cdot \sin\varphi \right)\frac{1}{\sqrt{{\left(\frac{a}{\rho }\right)}^{2}+1}}dS,$$

(64) $${\left(N_{M\varphi }\rho +F_{TR\varphi }\rho \right)}_{P}=\left(-a-k_{TR}\rho \right)\frac{\gamma \cdot g\left(\rho_{к1}-D-\;\rho \cdot \sin\varphi \right)dS}{\sqrt{{\left(\frac{a}{\rho }\right)}^{2}+1}}.$$

Using Equation (2), we obtain:

(65) $$M_{VRP}=\underset{\left(S\right)}{\iint }\left(a+k_{TR}\rho \right)\;\frac{\gamma \cdot g\left(\rho_{к1}-D-\;\rho \cdot \sin\varphi \right)dS}{\sqrt{{\left(\frac{a}{\rho }\right)}^{2}+1}}.$$

Taking into account that the elementary area is $dS=\sqrt{a^{2}+\rho^{2}}d\rho d\varphi$, the integral over the surface will take the form:

(66) $$M_{VRP}=\int_{\varphi_{0}}^{\varphi_{k}}\left(\int_{\rho_{0}}^{\rho_{к}}\left(a+k_{TR}\rho \right)\;\frac{\gamma \cdot g\left(\rho_{к1}-D-\;\rho \cdot \sin\varphi \right)}{\sqrt{{\left(\frac{a}{\rho }\right)}^{2}+1}}\sqrt{a^{2}+\rho^{2}}d\rho \right)d\varphi .$$

We also determine the contribution to the torque of the third component in Equation (51):

(67) $${\left(N_{M\varphi }\rho +F_{TR\varphi }\rho \right)}_{C}=-\lambda_{c}a-k_{TR}\lambda_{c}=\left(-a-k_{TR}\rho \right)\frac{g\cdot \gamma \cdot h\cdot k_{c}\frac{\left(\rho_{к1}-D-\rho \cdot \sin\varphi \right)}{l_{cp}}\cdot dS}{\left|grad\left(f\right)\right|};$$

(68) $${\left(N_{M\varphi }\rho +F_{TR\varphi }\rho \right)}_{C}=\left(-a-k_{TR}\rho \right)\frac{g\cdot \gamma \cdot h\cdot k_{c}\frac{\left(\rho_{к1}-D-\rho \cdot \sin\varphi \right)}{l_{cp}}\cdot dS}{\sqrt{{\left(\frac{a}{\rho }\right)}^{2}+1}}.$$

When it is summed into Equation (4), this component will be represented by an integral over the coordinates φ and ρ:

(69) $$M_{VRC}=\int_{\varphi_{0}}^{\varphi_{k}}(\int_{\rho_{0}}^{\rho_{k}}\left(a+k_{TR}\rho \right)\frac{g\cdot \gamma \cdot h\cdot k_{c}\frac{\left(\rho_{к1}-D-\rho \cdot \sin\varphi \right)}{l_{cp}}}{\sqrt{{\left(\frac{a}{\rho }\right)}^{2}+1}}\sqrt{a^{2}+\rho^{2}}d\rho )d\varphi .$$

Taking into account that the boundaries ρ and φ in each of the integrals do not depend on each other and are constants, after we are calculate the integrals over one variable, we determine their difference when substituting the boundaries of another one, which was considered constant while integrating. 

Thus, the torque is equal to the sum of three components:

(70) $$M_{VR}=M_{VRQ}+M_{VRP}+M_{VRC}.$$

If the values for the vectorial angles $\varphi_{0},\;\varphi_{k}$ are substituted into the limits of integration within one turn (the difference between them will be less than 2π, since the mixer is not fully loaded), and then the result is multiplied by the number of turns, then we get the total torque. The mixer can have screw surfaces with different directions. The calculations are given for the right screw, but Equation (62) is also valid for the left screw, although the axial response of the material to the screw (auger) will change its direction. Similarly, it is possible to sum up the torques from several screw surfaces with different pitch coefficients a and different ranges of the radius $\rho_{0},\rho_{k}$, which are fixed on the same shaft.

The value of power demand can be calculated using the following equation:

(71) $$P=\omega \cdot M_{VR}$$

## 4. Results and Discussion

### 4.1. The Effect of Structural and Technological Parameters on Power Consumption on the Basis of the Theoretical Model

Based on the presented calculations, theoretical study of the effect of structural and technological parameters on power consumption by the mixer motor was carried out. The nature of change of the surfaces, their appearance and the direction of the vector of change in the power value are the same for all three mixer screws, therefore, the surfaces obtained for the middle screw are given as an example. Analysis of the results is given for all three calculated components according to Equations (62), (66) and (69) included in Equation (51) and their aggregate capacity (Equation (70)).

**Figure 9:**
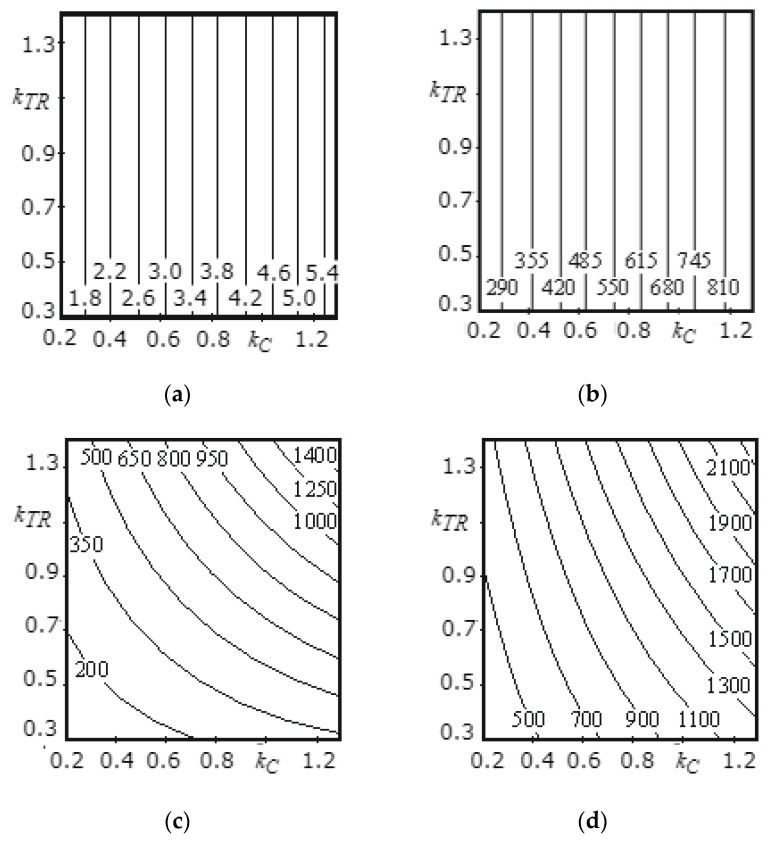
Dependence of the power value on the material coefficient of friction against steel and a material against material for the middle screw: (**a**) the surface constructed based on calculations of the second component; (**b**) the surface constructed based on calculations on the second component; (**c**) the surface constructed based on calculations on the third component; (**d**) the surface representing the change in the total power value for all three components.

**Figure 10:**
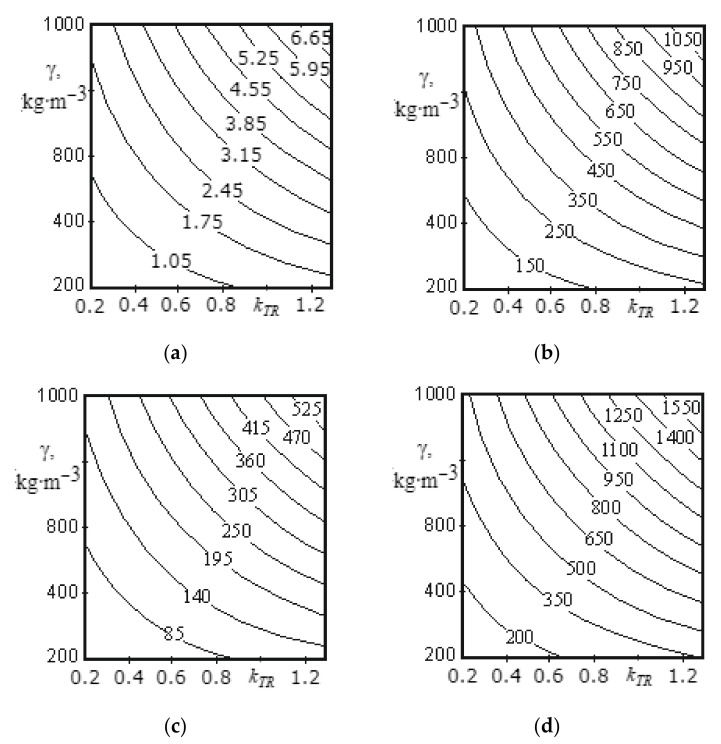
Dependence of the power value on the friction coefficient of the material against steel and on the material density for the middle screw: (**a**) the surface constructed based on calculations on the first component; (**b**) the surface constructed based on calculations on the second component; (**c**) the surface constructed based on calculations on the third component; (**d**) the surface representing the change in the total power value for all three components.

**Figure 11:**
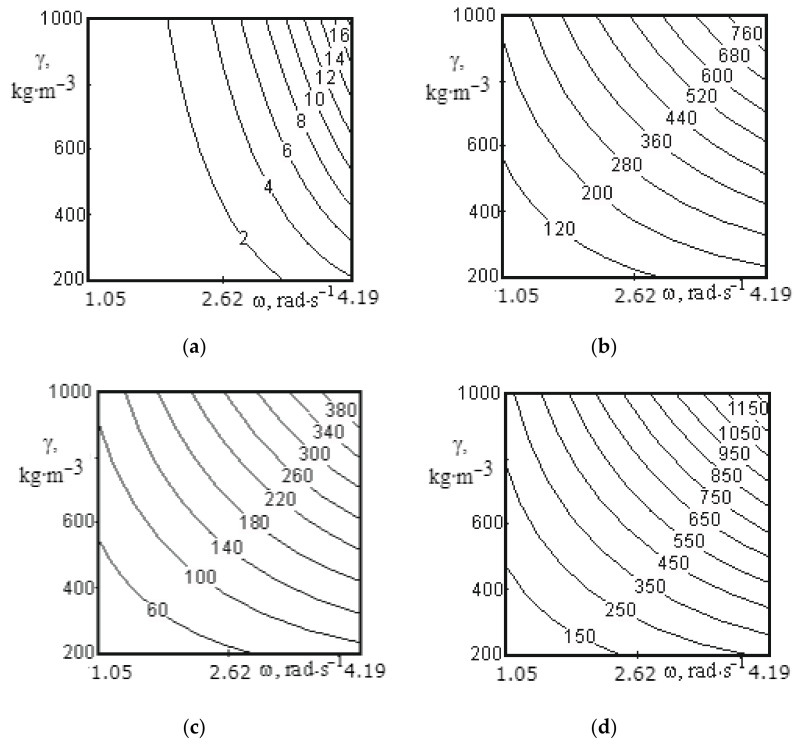
Dependence of the power value on the speed of mixer shaft rotation and the material density for the middle screw: (**a**) the surface constructed based on calculations on the first component; (**b**) the surface constructed based on calculations on the second component; (**c**) the surface constructed based on calculations on the third component; (**d**) the surface representing the change in the total power value for all three components.

**Figure 12:**
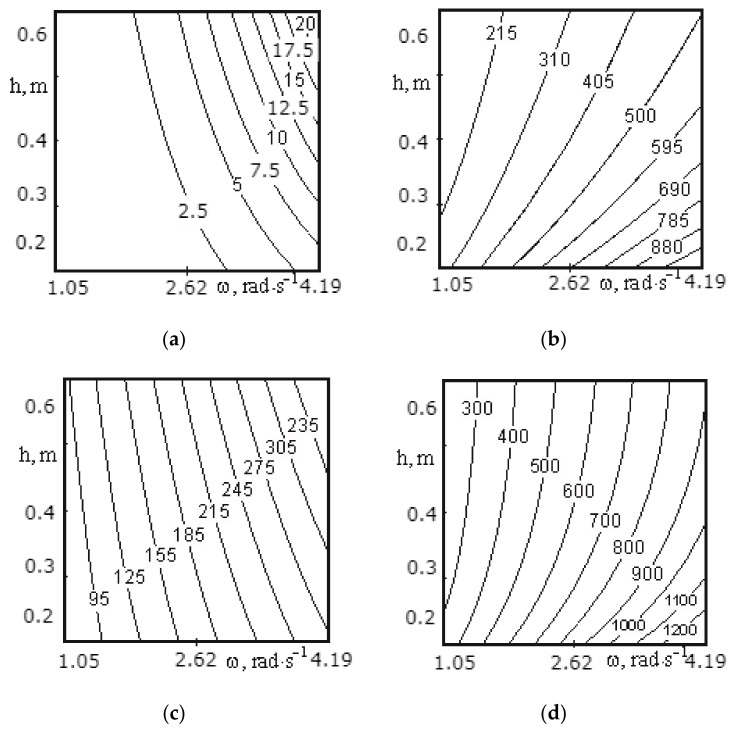
Dependence of the power value on the speed of mixer shaft rotation and the pitch of a screw tape turn for the middle screw: (**a**) the surface constructed based on calculations on the first component; (**b**) the surface constructed based on calculations on the second component; (**c**) the surface constructed based on calculations on the third component; (**d**) the surface representing the change in the total power value for all three components.

**Figure 13:**
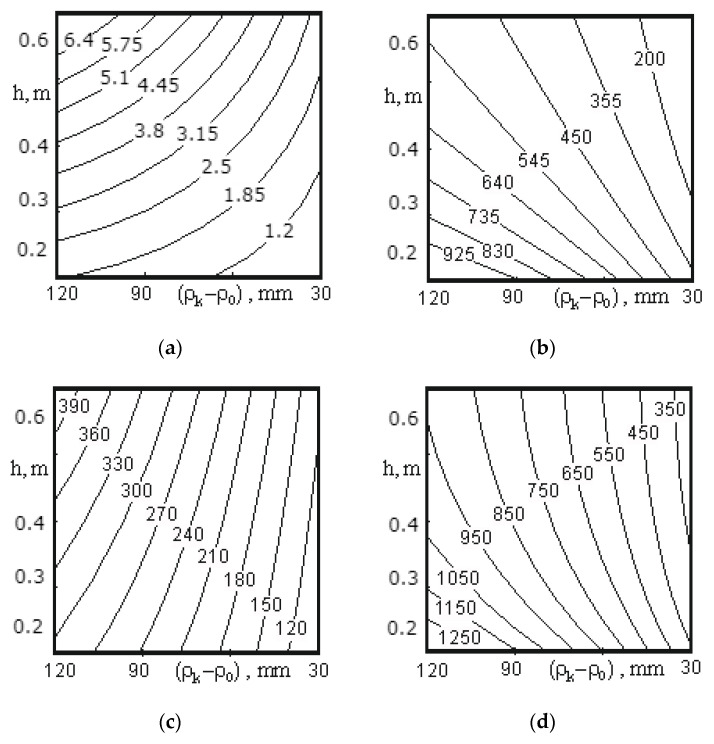
Dependence of the power value on the tape width of the screw and its pitch for the middle screw: (**a**) the surface constructed based on calculations on the first component; (**b**) the surface constructed based on calculations on the second component; (**c**) the surface constructed based on calculations on the third component; (**d**) the surface representing the change in the total power value for all three components.

**Figure 14:**
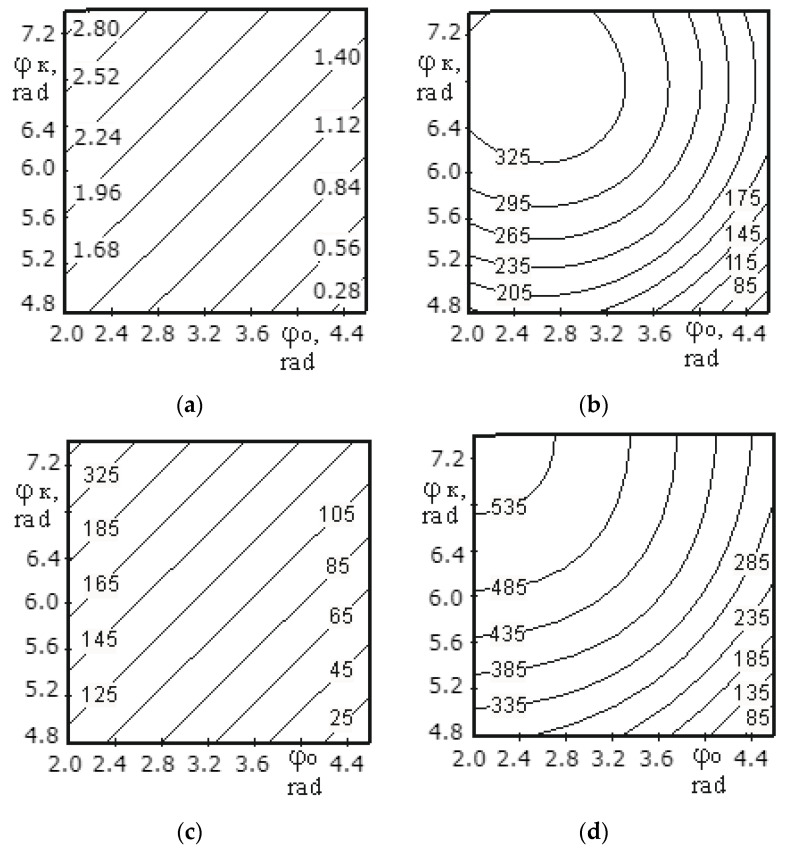
Dependences of the power value on the degree of mixer loading for the middle screw: (**a**) the surface constructed based on calculations on the first component; (**b**) the surface constructed based on calculations on the second component; (**c**) the surface constructed based on calculations on the third component; (**d**) the surface representing the change in the total power value for all three components.

Figure 9a, Figure 10a, Figure 11a, Figure 12a, Figure 13a and Figure 14a show a surface made according to the first component (which is described by Equation (62)), which takes into account the power used to accelerate the material from zero to nominal value. Figure 9b, Figure 10b, Figure 11b, Figure 12b, Figure 13b and Figure 14b demonstrate a surface constructed on the second component (according to Equation (66)), which takes into account the friction force of the material against the screw with regard to the loading height of the mixing chamber. Figure 9c, Figure 10c, Figure 11c, Figure 12c, Figure 13c and Figure 14c show a surface constructed on the third component (according to Equation (69)), which takes into account the friction force of the materials layers against each other, with regard to the loading height of the mixing chamber. The surfaces presented in Figure 9d, Figure 10d, Figure 11d, Figure 12d, Figure 13d and Figure 14d, constructed in result of the sum of all three components (according to Equation (70)), show the total effect of all factors on the three components by the power value, depending on the level of the mixer loading.

Figure 9 shows the surfaces reflecting the change in power from 285.4 W to 2385.2 W, depending on the coefficient of friction of the material against steel (0.2–1.3) and material against material (0.3–0.4), as well as the calculation method (component). The surface constructed on the first component (Figure 9a) and surface constructed on the second component (Figure 9b) are flat surfaces inclined at an angle to the horizontal surface, since the formula used for the first and second calculation methods does not take into account the friction of material against material, and the change in the coefficient of friction does not affect the calculated power parameters. The surface constructed on the third component (Figure 9c) has a curvilinear shape, since this calculation method takes into account both the friction of material against material when in motion, and the friction of material against steel surface of the screw. The surface constructed as a result of the sum of all three components (Figure 9d) has a curvilinear shape.

Analysis of Figure 9 shows that the change in the coefficient of friction of material against material from 0.3 to 1.4 has a lesser effect on the change of the power value (from 285.4 W to 690 W at *k_TP_* = 0.3 and from 1200 W to 2385.2 W at *k_TP_* = 1.3) than the change in the coefficient of friction of material against the screw surface from 0.3 to 1.3 (from 285.4 W to 1200 W at *k_C_* = 0.3 and from 690 W to 2385. 2 W at *k_C_* = 1.4). As a result of studies, it can be concluded that the more the mixture components are ground, the more power is consumed for their mixing.

Figure 10 shows surfaces that reflect the change in power from 80.3 to 1837.4 W, depending on coefficient of friction of the material against steel (0.2–1.3) and the material density (200–1000 kg/m^3^). The presented surfaces (Figure 10a–d) have a curvilinear shape.

Analysis of the surface shown in Figure 10d allows us to conclude that at a low material density (200–250 kg/m^3^), with a change in the friction coefficient from 0.2 to 1.3, the power consumption increases from 80. 3 to 350 W, and at a high material density (900-1000 kg/m^3^) the level of power consumption increases from 490 to 1837.4 W. With an increase in material density from 200 to 1000 kg/m^3^, the power value changes from 80.3 to 490 W (at a friction coefficient of 0.2) and from 350 to 1837.4 W (at a friction coefficient of 1.3). Based on the analysis of the study results presented in Figure 10d), we can say that the greater the friction coefficient and the higher the material density, the more power is consumed. Thus, the material with a more developed surface requires more power for mixing.

Figure 11 shows the surfaces reflecting the change in power from 60.06 to 1288.5 W depending on the speed of mixer shaft rotation (1.05–4.19 rad/s), material density (200–1000 kg/m^3^) and the component based on which calculations were made.

Analysis of surfaces presented in Figure 11 allows us to draw the following conclusions. Presented lines in the surfaces have nonlinear shape. According to Figure 11a, the change in the shaft speed from 1.05 to 4.19 rad/s leads to an increase in power from 0.07 to 3.6 W at a density of 200 kg/m^3^, and the change in speed from 1.05 to 4.19 rad/s increases the power value from 0.07 to 18.5 W at a material density of 1000 kg/m^3^. The change in the power value on the speed of rotation at a fixed density value has a parabolic relation in this case. Analysis of the surfaces constructed on other components (Figure 11b,c) points out that changes in the values of speed of rotation and density of material have the same effect on the nature of the change in the value of power.

For all surfaces in Figure 11, the power value reaches its minimum level at a speed of mixer shaft rotation of 1.05 rad/s and a density of 200 kg/m^3^, and its maximum level at a speed of mixer shaft rotation of 4.19 rad/s and a density of 1000 kg/m^3^. Thus, analysing the surfaces presented in Figure 11, we can conclude that the speed of the mixer shaft rotation should not exceed 2.62 rad/s and the mixed material should not be over-ground. At a shaft rotation speed of more than 2.62 rad/s, energy consumption significantly increases and there is practically no material movement along the mixer shaft axis, while at a rotation speed of 1.57 rad/s, the intensity of mixing of the components significantly decreases, which affects the quality of mixing, despite the reduction in energy consumption.

Figure 12 shows the surfaces reflecting the change in power from 180.09 to 1462.7 W depending on the speed of mixer shaft rotation (1.05–4.19 rad/s) and the pitch of a tape turn (0.2–0.6 m) for the medium screw. The surfaces constructed on the first (Figure 12a) and third component (Figure 12c) show the maximum power consumption at the pitch of a screw tape turn of 0.6 m and a speed of mixer shaft rotation of 4.19 rad/s, and the surface constructed on the second component (Figure 12b) reaches power at a pitch of 0.2 m and a speed of 4.19 rad/s. This is due to the fact that the second component takes into account the friction force of the material against the screw surface, thus, the more screw turns per unit length, the greater the friction force. The first and third components (Equations (62) and (66)) also take into account the friction force of the material against the screw surface, but based on the calculation method, its role is not so significant and does not exceed the effect of the rotation speed. Additionally, since the values obtained in the calculations on the second component significantly exceed the values obtained in the calculations for the first and third components, the shape of the surface constructed as a result of the sum of the values for all three components (see Figure 12d) follows the shape of the surface presented in Figure 12b.

A change in the screw pitch from 0.2 to 0.6 m with an increase in the speed of rotation from 1.05 to 2.09 rad/s has practically no effect on the change in the power value, and for the total values at 2.09 rad/s, the change does not exceed 150 W (see Figure 12).

Thus, the following conclusion can be drawn. The speed of mixer shaft rotation should not exceed 2.09 rad/s because the amount of power consumed by the motor has a square-law dependence on the rotational speed, and the screw pitch should be as large as possible. However, taking into account the quality of mixing of the material and the value of power consumption, the pitch of the middle screw should be in the range from 0.4 to 0.6 m.

Figure 13 presents the surfaces reflecting the change in power depending on the width of the screw tape and its pitch. At the same time, an inverse change in the power value calculated on the second component (Figure 13b) is observed, as opposed to the calculations on the first and third components (Figure 13a,b), respectively.

Analysis of the surface presented in Figure 13a allows to make the following conclusions. A change in the screw tape width from 30 to 120 mm at a pitch of 0.2 m has an insignificant effect on the increase in power (from 0.55 to 1.9 W), while at a pitch of 0.6 m, the power value changes from 2.4 to 9.5 W. This owes to the fact that the speed of axial movement of the material at a pitch of 0.6 m is greater, so the energy consumed to accelerate a particle will be of greater importance. The power value reaches its minimum value at a pitch of 0.2 m and a tape width of 30 mm, and the maximum value—at a pitch of 0.6 m and a tape width of 120 mm. As the tape width increases, both the amount of material transported and, accordingly, the power value also increases.

Examining the curves presented in Figure 13b the following conclusions can be made. The minimum power value can be obtained at a pitch of 0.6 m and a tape width of 30 mm, while the maximum value—at a pitch of 0.2 m and a tape width of 120 mm. A change in the tape width from 30 to 120 mm at a pitch of 0.6 m insignificantly affects the increase in power (from 165 to 525 W), and at a pitch of 0.2 m a more significant change occurs (from 360 to 1115 W). These changes are due to the fact that with a decrease in the screw pitch and an increase in the tape width, the size of the contact area between the screw tape and the material increases, which increases the friction force underlying the calculation on the second component, and, accordingly, the drive power.

Analysis of the surface constructed on the third component (Figure 13c) allows one to draw a conclusion that the minimum power value can be achieved at a pitch of the screw tape of 0.2 m and a tape width of 30 mm, while the maximum—at a pitch of 0.6 m and a tape width of 120 mm. A change in the tape width from 30 mm to 120 mm has a more significant effect on the power value (from 130 to 400 W at a pitch of 0.6 m) than a change in the pitch from 0.2 m to 0.6 m (from 290 to 400 W at a tape width of 120 mm). This owes to the fact that with an increase in the tape width the amount of material transported by one turn of the screw increases by a greater amount than with an increase in the screw pitch.

Analysis of surface in Figure 13d allows drawing the following conclusion. The pitch of the middle screw should be in the range of 0.4–0.6 m, with a tape width of 30 to 50 mm, which corresponds to the lowest energy consumption.

Figure 14 shows the surfaces reflecting the change in power (from 40.25 W to 592.9 W) depending on the mixer loading value (from 10 to 95%) and on the calculation method.

Based on the analysis of Figure 14, a higher power value of 360 W (when the mixer is loaded at 95%) is obtained when calculating power on the second component (surface presented in Figure 14 b)), which takes into account the friction force of the material against the screw, with regard to the loading height of the mixing chamber. The lowest power value of 2.9 W (when the mixer is loaded at 95%) is obtained when calculating power on the first component (surface presented in Figure 14a), taking into account the power consumed to give the material a speed from zero to nominal value.

The surface constructed on the third component (Figure 14c), which takes into account the friction force of the layers of material against each other, with regard to the loading height of the mixing chamber, occupies an average position between the first and second surfaces (Figure 14a,b respectively), and the power value is 230 W (when the mixer is loaded at 95%). Analysis of Figure 14 shows that the greater the mixer loading value, the more power is consumed; at the same time the dependences obtained on the first and third components have a rectilinear shape, while on the second one—a curvilinear shape.

### 4.2. Experimental Verification of Power Consumption Model

**Figure 15:**
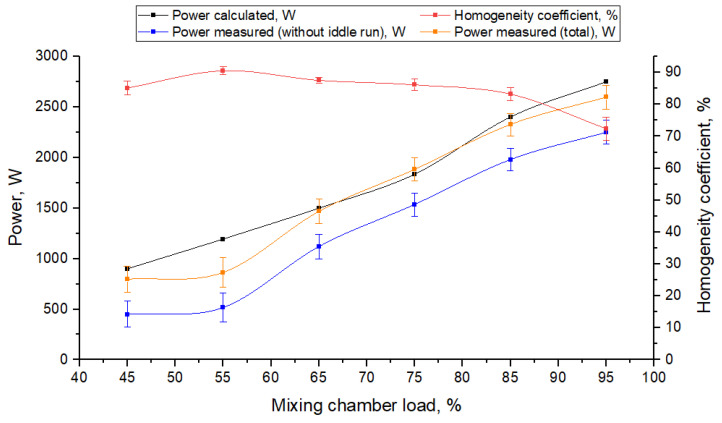
Mixer power consumption and mixture homogeneity coefficient in a function of the mixing chamber load.

In order to verify the power consumption model of mixing for the analysed screw mixer, an experiment, described in Section 2.3., involving mixing rye with barley in the presence of peas as a control material, was conducted. Figure 15 shows the results of power consumption tests excluding power for idle motion (blue curve), total power input (orange curve) and the feed material homogeneity coefficient (red curve) in a function of the mixing chamber load determined in the experiment. 

Additionally, when carrying out theoretical studies, the dependence of the power required for mixing the components of compound feed on the mixing chamber loading value is determined. When carrying out calculations, the angle of interaction of the material with each of the screws is taken into account. Based on the results of calculations, the dependence of the power and the amount of material in the mixer is shown in Figure 15 (black curve).

The analysis of homogeneity coefficient curve (red) presented in Figure 15 shows that the homogeneity coefficient of the finished product reaches its maximum value when the chamber is loaded at 55%, and it is 90.5% [3].

Based on the structural features and working conditions it has been found that the amount of material in a horizontal mixer cannot be smaller than 55% or larger than 75%. Hence, based on the analysis from Figure 15, taking into consideration quality of mixing, the amount of material in the mixing chamber should be 55–75%, while the homogeneity coefficient of the finished material varies from 90.5 to 86.1%, the power value is from 1194 to 1835 W (calculated), from 517 to 1535 W without power for idle run (obtained in experimental studies) and from 864 W to 1882 W of total power used for mixing. 

When assessing experimental values of power, the coefficient of variation is equal to 28%, when the mixing chamber is loaded at 55%, and 6.3%, when it is loaded at 75%. It is thought that the results are reliable and homogeneous if the variability coefficient does not exceed 33%, as it is in this case.

To provide the mixer with efficient operation the mixing chamber loading should be 65–75%. Comparing the results of power consumption theoretical analyses and experimental tests for this range of chamber loading, consistence of results must be confirmed (see Figure 15). Relative error (calculated according to dependence shown in Equation (72)) of the theoretically determined power values predicted for the mixer loading equal to 65% is in the range of 5.5–11.4% (taking into account the smallest and the largest measured value of the total power consumed by the mixer) and it is in the range 3.8–8.1% for 75% of the mixing chamber loading which allows to say that the developed theoretical model of power demand is effective in predicting the machine power consumption and can be used in practice. For the remaining levels of loading, the relative error for power consumption prediction did not exceed 15%, with the exception of loading equal to 45% (error for the lowest measured value of power was 34.6%) and 55%, for which the relative error was within the range of 18.4–66.0%, which can be caused by the highest scatter of experimental test results (variability coefficient equal to 28%):

(72) $$RE=\frac{\left|P_{T}-P_{E}\right|}{P_{E}}\cdot 100\%$$

where *P_T_* is the value of the power prediction based on calculations, W, and *P_E_* is the experimentally-determined value of power, W.

Thus, we can say that an increase in the mixer loading from 55 to 75% for the obtained mixture homogeneity coefficient being 86.1% (Figure 15), good enough to be used for feeding relevant animal species or poultry, making it possible to increase the mixer capacity from 2.36 to 3.21 t/h.

## 5. Conclusions

The mathematical dependencies obtained as a result of the carried-out theoretical studies make it possible to calculate the value of power consumed by the mixer shaft drive, taking into account various structural and technological factors.

A change in the value of the coefficient of friction of the material against material from 0.3 to 1.4 has a smaller effect on the change in the power value (from 285.4 W to 690 W at *k_TP_* = 0.3 and from 1200 W to 2385.2 W at *k_TP_* = 1.3) than the change in the coefficient of friction of the material against the screw surface from 0.3 to 1.3 (from 285.4 W to 1200 W at *k_C_* = 0.3 and from 690 W to 2385.2 W at *k_C_* = 1.4). Thus, the more the mixture components are ground, the more power is consumed for their mixing.

At a low material density (200–250 kg/m^3^), when the friction coefficient changes from 0.2 to 1.3, the power consumption increases from 80.3 to 350 W, and at a high material density (900–1000 kg/m^3^) the value of power consumption increases from 490 to 1837.4 W. With an increase in the material density from 200 to 1000 kg/m^3^, the power value changes from 80.3 to 490 W (at a friction coefficient of 0.2) and from 350 to 1837.4 W (at a friction coefficient of 1.3). Thus, the material with a more developed surface requires more power for mixing.

Analysing the effect of a change in the speed of mixer shaft rotation and the density on the power consumption, it can be concluded that the speed of mixer shaft rotation should not exceed 2.09 rad/s, since the value of power consumed by the motor has a square-law dependence on the rotational speed, and the mixed material should not be over-ground.

Evaluation of the effect of a change in the speed of mixer shaft rotation and the screw pitch on a change in the power value allows the following conclusion to be drawn. The speed of mixer screw shaft rotation should not exceed 2.09 rad/s and the tape pitch of the screw turn should be as large as possible, but taking into account the material mixing quality and the value of power consumption, the pitch of the middle screw should be in the range of 0.4–0.6 m.

Theoretical studies of the influence of the dependence of the power value on the width of the screw tape and its pitch allow to conclude that the pitch of the middle screw should be in the range of 0.4–0.6 m, with a tape width of 30–50 mm, which corresponds to the lowest energy consumption. The pitch of the external screw should be in the range of 0.3–0.5 m with a tape width of 30–70 mm, and for the internal screw, the pitch should be from 0.23 to 0.54 m with a tape width of 30–100 mm.

Based on the conducted tests and conditions of a horizontal mixer operation, the amount of material in the mixing chamber should range within 55–75%, as then the homogeneity coefficient of the obtained material ranges from 90.5 to 86.1%.

An increase in the mixer loading from 55 to 75% with a sufficient homogeneity coefficient of the resulting mixture of 86.1% for feeding the corresponding species of animals or poultry makes it possible to increase the mixer throughput from 2.36 to 3.21 t/h.

In the assumed interval 65–75% of the mixer loading that provides effective and efficient mixing, the results for power consumption determined experimentally and theoretically are consistent which is indicated by low value of relative errors of the results. Thus, it may be concluded that the conducted theoretical studies quite completely characterize the processes occurring in a horizontal tape mixer in the process of feed preparation.

Theoretical and practical significance of this study provides the possibility of using the results of this theoretical research in the mixer design stage for the determination of power consumption of working unit drives with regard to the structural and technical factors. Application of the developed mathematical models allows a reduction in the costs of design, manufacturing, and optimizes the operation of mixers with tape-working bodies. The model of power consumption, including many material, structural and processing variables, enables the improvement of the quality of animal feed mixtures, reduces energy consumption and facilitates their preparation.

Further research should be focused on the identification and investigation of the factors which have the largest impact on power consumption in the process of mixing when such structural and technological parameters as frequency—screw tape width; blade montage—and structural changes of the tape-screw mixing unit are altered.

## Figures and Tables

**Table 1:** Parameters of the stirrer used in a screw mixer.

Parameter	Symbol	Value	Unit
Diameter of external screw	*D*_1_	1	m
Diameter of middle screw	*D*_2_	0.75	m
Diameter of internal screw	*D*_3_	0.4	m
Diameter of internal edges of the external screw	*d*_1_	0.90	m
Diameter of internal edges of the middle screw	*d*_2_	0.65	m
Diameter of internal edges of the internal screw	*d*_3_	0.26	m
Pitch of external screw	*h*_1_	0.3	m
Pitch of middle screw	*h*_2_	0.4	m
Pitch of internal screw	*h*_3_	0.24	m
Width of the ribbon tape	*g*	0.05	m
Length of stirrer	*l*	1.8	m

**Table 2:** Material properties used in calculations.

Parameter	Symbol	Range (Value)*	Unit
Specific gravity of grain mixture [40,41]	γ	200–1000 (750)	kg·m^−3^
Coefficient of friction between material and steel [40,41]	*k_TR_*	0.2–1.3 (0.4)	-
Coefficient of friction between material grains [40,41]	*k_C_*	0.2–1.4 (0.37)	-

()* Values corresponding to a mixture of materials used in a physical experiment.

## Data Availability

Data sharing not applicable.
